# Uncommon disease with unusual presentation: A case report of acute necrotizing otitis media

**DOI:** 10.1016/j.ijscr.2022.107756

**Published:** 2022-10-21

**Authors:** Rula Naqi, Ahmed Khalid Ahmed, Hesham Yusuf Alrayyes

**Affiliations:** King Hamad University Hospital, Bahrain

**Keywords:** Otology, Otitis media, Otorhinolaryngology, ENT

## Abstract

**Introduction:**

Acute necrotizing otitis media (ANOM) is defined as a severe middle ear infection that results in tympanic membrane necrosis. Commonly caused by bacteria infection. It is a rare entity of otitis media, with unknown prevalence. Usually seen in immunocompromised patients.

**Case presentation:**

We present a case report of a 79-year-old diabetic male, who presented to the otolaryngology clinic with history of intermittent worsening nocturnal left ear pain. The clinical presentation was suggesting necrotizing otitis externa despite lacking the classical picture of external ear canal edema and granulation, in the presence of a dull bulging tympanic membrane. Then the patient developed hearing loss, along with ear discharge, high inflammatory markers, and significant imaging findings. This case represents an unusual presentation of ANOM. The patient responded well to aggressive medical treatment, along with grommet insertion.

**Conclusion:**

Despite the development of antibiotics, ANOM is still possible, especially in high-risk patient. A high index of suspicion, along with detailed history, physical examination, and supportive imaging are needed, to diagnose ANOM. Aggressive treatment with antibiotics should be started immediately, guided by the culture results, to improve the prognosis and prevent further complications.

## Highlights

• Acute necrotizing otitis media (ANOM) severe middle ear infection that results in tympanic membrane necrosis
• It is caused by bacterial infection and commonly seen in immunocompromised patients.
• It can present with ear pain, otorrhea, conductive hearing loss, or facial palsy [2, 6].
• Investigations done in ANOM includes blood tests for inflammatory markers and imaging, mainly CT and MRI
• The mainstay treatment of ANOM is antibiotics, both topical and intravenous

## 1 Introduction

Otitis media (OM) is a wide array of inflammatory disorders of the middle ear. It includes acute, recurrent, chronic otitis media, otitis media with effusion (OME), acute necrotizing otitis media (ANOM) and cholesteatoma [1].

ANOM is defined as a severe middle ear infection that results in tympanic cavity necrosis [2]. Mostly, ANOM is caused by bacteria, and the most common bacteria culture is beta-hemolytic streptococcus. ANOM is commonly seen in immunocompromised patients [3]. Although the incidence of otitis media (OM) is high, the prevalence of ANOM is unknown, as it is relatively rare. Compared to acute suppurative OM, ANOM had significantly reduced after the antibiotic era [2].

There are no specific diagnostic criteria for ANOM, which makes it a challenging disease, confounded more by the lack of clear management protocol.

## 2 Case report

This case report is in line with the SCARE 2020 [25].

A 79 year old male with a known case of diabetes mellitus, hypertension, chronic kidney disease, and atrial fibrillation presented to the otolaryngology clinic with history of intermittent left progressive nocturnal otalgia for the past 2 months. The pain was dull, localized, rated 5 out of 10 in severity. There was no associated active ear discharge, hearing loss, facial nerve palsy, or any nasal symptoms. Upon physical examination, the patient was vitally stable. Left ear examination showed a patent, erythematous ear canal, with no evidence of infection or granulation tissue. However, there was bulging of the tympanic membrane. The right ear was normal. Accordingly, the patient was started on oral moxifloxacin and ciprofloxacin for one week.

**Fig. 1:**
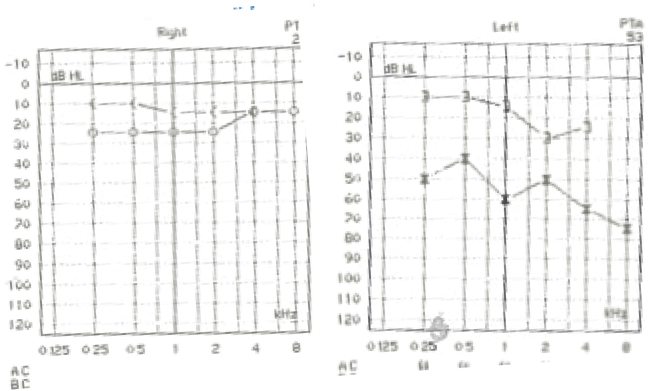
First pure tone audiometry done, showing normal right ear and conductive hearing loss in the left ear.

**Fig. 2:**
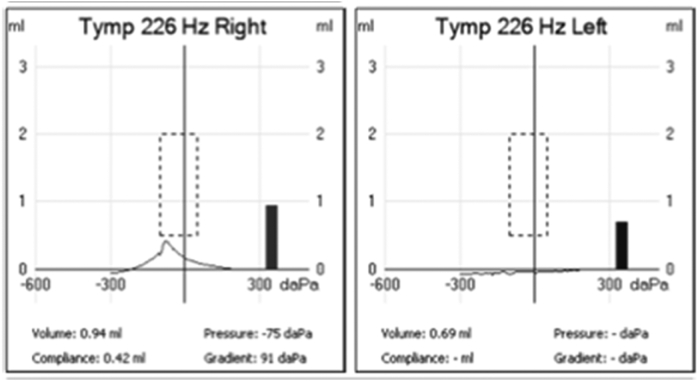
First tympanometry, showing type A in right ear, which is normal finding and type B in the left ear, which indicates fluid in the middle ear.

During follow up appointment, it was noted that the patient did not finish his antibiotics course because the pain improved. However, he noticed sudden onset hearing loss in the left ear. When examined, the left ear canal was normal, without erythema or swelling, but the tympanic membrane was dull. The right ear was completely normal. Tympanometry and pure tone audiometry were done (Fig. 1, Fig. 2), showing type B and conductive hearing loss respectively in the left ear, while the right ear was completely normal. Complete blood count (CBC) showed slightly elevated white blood cells (WBC) (12.6, the normal range is 4–11), with neutrophils predominance, and low hemoglobin (Hb) level. C-Reacive Protein (CRP) was high (38.24, the normal range is 0–10), erythrocyte sedimentation rate (ESR) was also high (120, the normal range is 0–20). At this stage, myringotomy and grommet insertion were done in the clinic for the left ear. One week later, grommet was in place and patient was doing well.

**Fig. 3:**
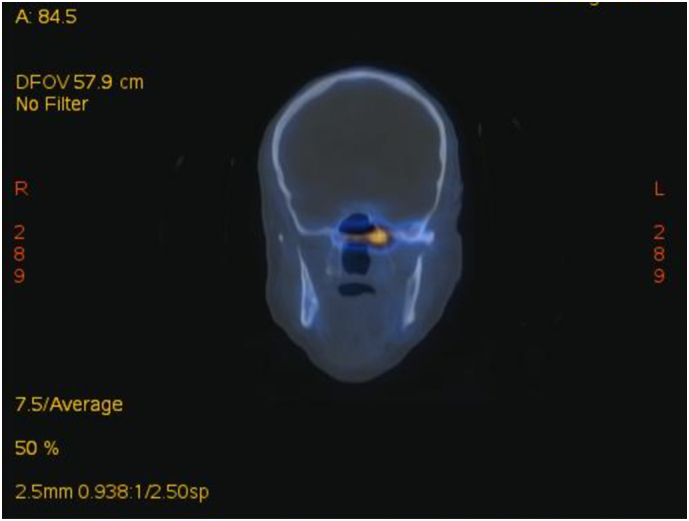
Bone scan showing scintigraphic findings are suggestive of bone remodeling of left middle ear bones extending to mastoid air cells and sphenoid bone suggestive of active bone infection.

After one month, the patient presented again to the clinic with left ear pain with intermittent discharge for two weeks duration. The discharge was yellow in color with no blood. There was no tinnitus, dizziness, or symptoms of facial nerve palsy. Left ear examination showed an inflamed ear canal with yellowish discharge. The tympanic membrane was dull. Ear swab culture showed no organisms. Patient was also referred for bone scan (Fig. 3). The scintigraphic findings of the scan were suggestive of bone remodeling of left middle ear bones extending to mastoid air cells and sphenoid bone suggestive of active bone infection. This indicated a necrotizing process originating from the ear.

Since the external ear looked normal, without edematous changes, swelling, granulation tissue, and most importantly, pain upon ear examination, necrotizing otitis externa was unlikely. Meanwhile, there was a suspicion of the condition being that of necrotizing otitis media, especially because the patient was having active ear discharge, along with bone invasion. Additionally, the inflammatory markers were persistently high. The CRP increased to 52.07, the WBC increased from 11.3 to 12.3 (the normal range 4–11) and the ESR increased to 85.

Although necrotizing otitis media is not a common disease, it was suspected in this case, due to the patient age, comorbidities, namely diabetes, and due to progression of symptoms. Since the patient was vitally stable and looking well, aggressive oral antibiotic course was the treatment of choice. Patient was started on ciprofloxacin and ofloxacin, and tetrahydrozoline.

**Fig. 4:**
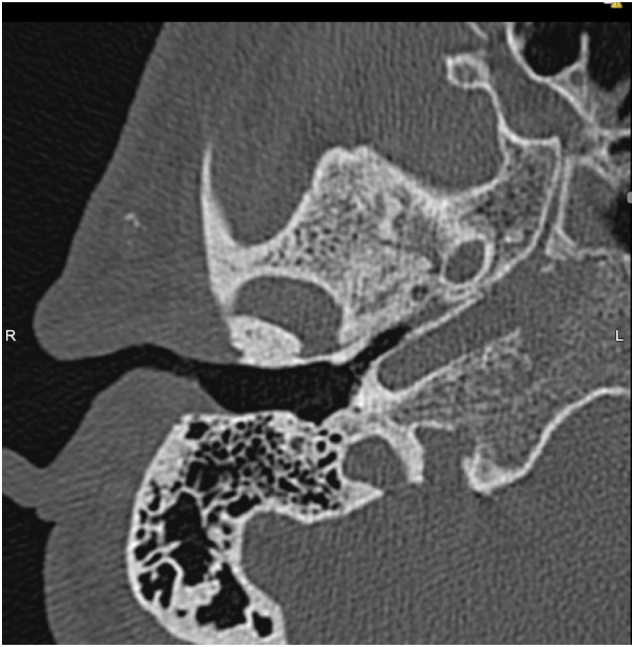
Fused PET/CT image showing disease progression regarding the extension of the active bone infection involving the left side of the skull base bones to currently involve the sphenoid body and right petrous with currently detected hypermetabolic nasopharyngeal mucosal thickening and hypermetabolic activity at prevertebral space and right carotid space.

One week later, patient was still having left ear pain, but the discharge reduced significantly. Therefore, patient was sent for further imaging. Fused positron emission tomography (PET)/computed tomography (CT) image showed progression of the active bone infection, compared with the previous bone scan. There was involvement of the left side of the skull base bones to currently involve the sphenoid body and right petrous with currently detected hypermetabolic nasopharyngeal mucosal thickening and hypermetabolic activity at prevertebral space and right carotid space (Fig. 4).

**Fig. 5:**
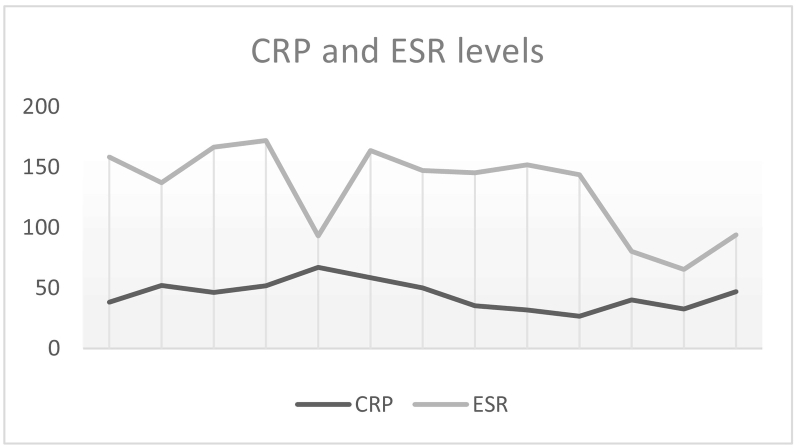
This graph represents the fluctuation in CRP and ESR levels, from the first visit until the end of the inpatient admission period. Both CRP and ESR were elevated initially, and both curved downward at the end period.

Patient was admitted immediately to a tertiary center and was started on intravenous (IV) ceftazidime antibiotic course and topical ofloxacin antibiotics. ESR and CRP were continuously monitored during admission as shown (Fig. 5).

Upon discharge, he completed a six weeks course of IV ceftazidime and topical ofloxacin. The pain improved, but the hearing loss did not. Otherwise, the patient was stable. He was discharged home on topical ofloxacin.

**Fig. 6:**
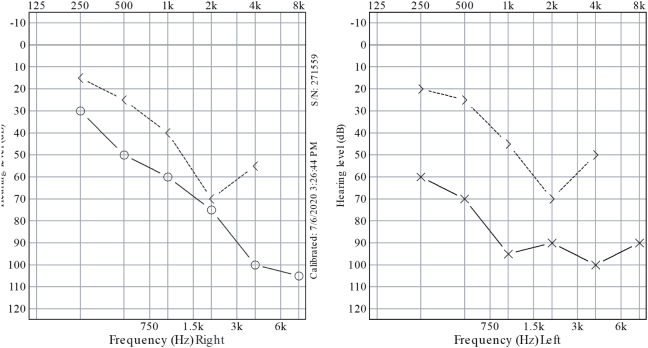
Second pure tone audiometry, which is done after discharge. There is sensorineural hearing loss in the right ear. In the left ear there is mixed sensorineural and conductive hearing loss.

**Fig. 7:**
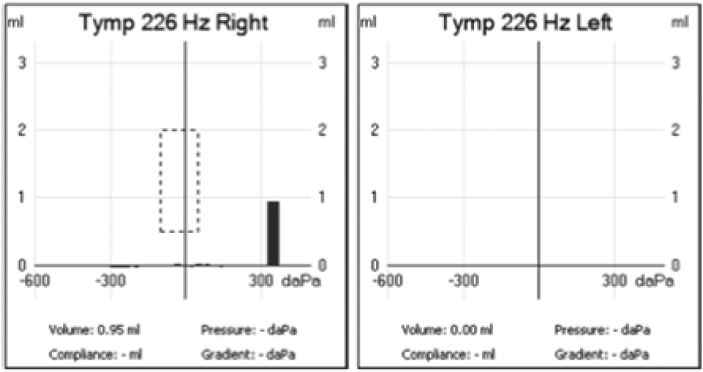
Second tympanometry, which is done after discharge. In the left ear, there is no reading, because the patient has grommet. In the right ear, it is type B.

One month later, the patient was seen in the otolaryngology clinic again. He was doing well. The left ear pain and otorrhea had completely resolved. However, he was still complaining of hearing loss and recently developed tinnitus. Audiometry and tympanometry were repeated. (Fig. 6, Fig. 7) The patient developed mixed hearing loss in the left ear and sensorineural hearing loss in the right ear. Patient was advised to use hearing aid to improve the hearing and eventually the tinnitus will improve as well.

## 3 Discussion

### 3.1 Incidence of ANOM

Infectious disease medicine highly evolved because of the development of vaccines and antibiotics [4]. Some diseases were eradicated, like polio, and others had dramatically reduced, like ANOM [3], [5]. In literature, the incidence of ANOM is not reported, and there are only around 3 cases published. These cases were within different generations, ranging from 1986 to 2020 [2], [3], [6].

### 3.2 Definition of ANOM

ANOM is an aggressive infection of the middle ear cavity that result in destruction of cavity's content, along with necrosis and sloughing of the tympanic cavity [2], [6]. In our case, initially the patient had only symptoms involving the external ear canal and normal looking tympanic membrane, pointing towards otitis externa. After using the antibiotics, the external canal returned to normal, but the tympanic membrane became dull. However, the was no perforation or any necrotic changes seen within the tympanic membrane.

### 3.3 Epidemiology of ANOM

ANOM is more likely to be encountered in immunocompromised patients. This includes infants or young children who are critically ill with scarlet fever and pneumonia. It is also seen in elderly patients with comorbidities, such as diabetes mellitus, as seen in our case [2], [7].

### 3.4 Pathophysiology of ANOM

ANOM is known to be caused by bacterial infection, as mentioned previously, with beta-hemolytic *Streptococcus* being the commonest organism [3]. The proposed pathophysiology of ANOM is related to the exotoxins, superantigens, and proteases with cytolytic function molecules, that are produced by the bacteria. They are known to cause necrosis of any body tissue involved, along with the systemic toxicity [3], [8]. However, in our case, ear swab was taken for culture and no organism was found. This is an unusual finding compared to the other reported cases [2], [3], [6]. It can be justified by the fact that the patient was initially diagnosed as otitis externa and was started on a course of antibiotics. Usually, antibiotics interfere with the culture findings [9].

### 3.5 Clinical findings of ANOM

Since many structures lie within the middle ear, patients with ANOM can present with different symptoms. The most common presentation is ear pain with sudden onset otorrhea, which is similar to otitis externa [3], [10]. Patients can develop conductive hearing loss, due to tympanic membrane perforation or ossicles involvement [3]. In some cases reported, patient with ANOM had signs and symptoms of facial palsy [2], [6].

Physical examination of the ear may show middle ear cavity full of granulation tissue without landmarks and necrosis or perforation within the tympanic membrane [3]. If ANOM was associated with necrotizing otitis externa, the auditory canal will be inflamed and edematous, blocking the view to the tympanic membrane and structures behind [2], [11], [12]. This can delay or even mask the diagnosis of ANOM.

In our case, the patient had an unusual presentation of ANOM, as his symptoms and signs were involving the external auditory canal mainly. Later on, he presented with hearing loss and ear discharge which points towards the middle ear.

Since the initial examination was done using otoscopy, there is always a chance to miss middle ear diseases, especially if the light output was suboptimal [13]. Microscopy and endoscopy are much better in visualizing the tympanic membrane and middle ear structures [14], [15].

Moreover, there is a possibility that the necrotizing process arose initially from the external ear, expanding into the middle ear especially that a grommet was inserted. Furthermore, as the patient did not complete the antibiotics course for the otitis externa, this could have created a port of the infection to spread into the middle ear, causing ANOM. However, the fact that the patient had localized pain, with mild erythematous changes, without swelling and without granulation tissue, physicians were able to visualize the tympanic membrane, making necrotizing otitis externa unlikely. Additionally, these symptoms resolved within less than a week of antibiotics. Usually, necrotizing otitis externa cases are treated aggressively for weeks [12].

The only way to know the exact answer is to get exposure to many cases of ANOM [2]. The fact that ANOM is now a rare disease makes it more difficult [2]. Having many cases of ANOM reported, understanding them, the symptoms, signs, and taking into consideration the epidemiology and comorbidities associated with each case will help in exploring ANOM. This is the main aim of this case report.

### 3.6 Investigations of ANOM

The aim of the investigations is to confirm ANOM diagnoses and to assess the degree of tissue involvement. Firstly, blood sample taken to measure CRP, ESR and WBC with differential are important markers from diagnostic and prognostic point of view [17]. In our case, CRP and ESR were monitored regularly before and during the admission, to assess the prognosis and the improvement of the disease, as seen in Fig. 5. CRP is known to rise immediately with the onset of inflammatory processes a curves down within a week. ESR increases much slower and remains high for a longer period [18]. Having both CRP and ESR elevated confirms the ongoing inflammatory state (Fig. 5).

Secondly, pure tone audiometry reveals the degree and the type of hearing loss [19]. Moderate conductive hearing loss is mostly seen; however profound sensorineural hearing loss is still a possibility [3]. Our patient had a conductive hearing loss, as seen in (Fig. 1). However, even after aggressive antibiotic treatment, the hearing did not improve. Unfortunately, it had worsened, along with bilateral sensorineural hearing loss (Fig. 6). This can be explained by the effect ceftazidime, which has ototoxic effect [21]. Also, age and comorbidities could have played a role in hearing loss, resulting in presbycusis. However, the fact that it developed in short period makes it unlikely [22]. In addition, it could be Idiopathic sudden sensorineural hearing loss, which is defined as “sensorineural hearing loss of 30 decibels (dB) or more over at least three contiguous audiometric frequencies with an onset of fewer than three days.” [23].

Conversely, in one of the reported cases, a young healthy male patient had reversible hearing loss after ANOM [3]. The hearing loss was sensorineural type, and it was justified by the impact of bacterial exotoxin on the cochlea. As the disease resolved, the exotoxin effect disappeared, and the hearing loss was reversed back to normal [3].

Thirdly, ear swab with microscopy culture and sensitivity will help in the management plan and antibiotic choice. If no organism is detected, a biopsy should be considered [6]. In our case, the ear swab culture showed no organism, as discussed previously. This could be explained by the fact that the patient received an antibiotic course previously [9].

Finally, imaging should be done, as ANOM can erode into the nearby tissue, including external auditory canal, skull base, cranial cavity, and into the central nervous system [2]. Bone scan is a type of imaging that assess radionuclide tracer accumulation, technetium (Tc 99m), within active osteoblasts [20]. It helps in initial assessment for the degree and extent of bone invasion [12], [20]. This should be followed by Magnetic resonance imaging (MRI) or Computerized tomography (CT) scan of the head [2]. The CT scan is good in detecting bony lesions, involvement of fat in subtemporal spaces and demineralization and destruction of mastoid cortex [12]. MRI, on the other hand, provides detailed anatomy of parts involved and assesses the degree of soft tissue invasion [12]. Gallium scan, which is a positron-emitting isotope, used in assessing variable inflammatory and malignant diseases, including ANOM [24]. Nevertheless, all imaging modalities shows sites of damage but they do not differentiate between active and resolving diseases [12].

### 3.7 Treatment of ANOM

The mainstay treatment of ANOM is antibiotics, both topical and intravenous [2], [3]. Usually penicillin is first choice. Alternative options can be considered according to the culture sensitivity and patient's allergy [3]. Surgical debridement is reserved for refractory cases, or if there is temporal bone involvement [2]. The prognosis of ANOM is mainly dependent on the degree of invasion [3]. However, there are no accurate cut off points to guide the treatment.

In our case, the patient had significant bone invasion including the skull base bones, sphenoid body, and right petrous bone. Despite that, he was treated medically. He received a six-week course of IV ceftazidime and topical ofloxacin. No surgical intervention was needed as the patient improved afterwards. As for the hearing loss, it is probably age related, the patient was advised to use hearing aid.

## 4 Conclusion

Despite the development of antibiotics, ANOM is still possible, especially in high-risk patient [2]. A high index of suspicion, along with detailed history, physical examination, and supportive imaging are needed, to diagnose ANOM [3]. Even if the disease was masked by other comorbidities, having a holistic approach to patients, along with regular follow up, will eventually reveal the hidden [4]. Most importantly, aggressive treatment with IV or oral antibiotics should be started immediately in ANOM, guided by the culture results, to improve the prognosis and prevent further complications [2]. If antibiotics were not enough, surgical debridement would be the second option [2]. More case reports about ANOM are needed to understand the exact pathophysiology and to create treatment guidelines.

## Funding

This research did not receive any specific grant from funding agencies in the public, commercial, or not-for-profit sectors.

## Ethical approval

This case report is exempt from ethical approval at our institution.

## Consent

Written informed consent was obtained from the patient for publication of this case report and accompanying images. A copy of the written consent is available for review by the Editor-in-Chief of this journal on request.

## Registration of research studies

Not applicable.

## Guarantor

Professor Hesham Yusuf Alrayyes.

## Provenance and peer review

Not commissioned, externally peer-reviewed.

## Declaration of competing interest

The authors state that they have no conflicts of interest for this report.
